# Supplementary material for: A Critical View of the Application of the APEX Software (Aqueous Photochemistry of Environmentally-Occurring Xenobiotics) to Predict Photoreaction Kinetics in Surface Freshwaters
Source: Molecules. 2019 Dec 18;25(1):9. doi: 10.3390/molecules25010009 (PMC7017383; doi:10.3390/molecules25010009)
Supplement: Supplementary file 1 [file molecules-25-00009-s001.zip › 3.2.4_gcc-4.4.0.zip!/3.2.4_gcc-4.4.0/3.2.4_gcc-4.4.0/doc/octave/html/interpreter/Function-Handles-Inline-Functions-and-Anonymous-Functions.html]

Function Handles Inline Functions and Anonymous Functions - Untitled


Next: Commands,
Previous: Script Files,
Up: Functions and Scripts


---

### 11.9 Function Handles, Inline Functions, and Anonymous Functions

It can be very convenient store a function in a variable so that it
can be passed to a different function. For example, a function that
performs numerical minimization needs access to the function that
should be minimized.

- Function Handles- Anonymous Functions- Inline Functions
